# Equity in access through a digital hospital appointment system: a reflexive thematic analysis of frontline registration staff perspectives in Türkiye

**DOI:** 10.3389/fpubh.2026.1958637

**Published:** 2026-10-09

**Authors:** Habibullah Akıncı, Sema Dalkılıç

**Affiliations:** 1 Department of Social Work, Faculty of Health Sciences, Yozgat Bozok University, Yozgat, Türkiye; 2 Department of Health Management, Faculty of Economics and Administrative Sciences, Yozgat Bozok University, Yozgat, Türkiye

**Keywords:** digital health equity, hospital appointment systems, healthcare access, street-level bureaucracy, reduced inequalities (SDG10)

## Abstract

**Introduction:**

Digital appointment systems increasingly mediate access to healthcare. However, not all individuals benefit equally from these systems, and the experiences of the frontline staff responsible for operating them remain underexplored. This qualitative study examined how hospital registration staff perceive access inequities, exercise discretion when formal procedures fail to meet patients’ needs, and understand equality and equity.

**Methods:**

Semi-structured interviews were conducted with 20 frontline registration staff working in two public hospitals in Central Anatolia, Türkiye. Data were analyzed using reflexive thematic analysis, combining semantic and latent interpretation. Coding was conducted primarily in a data-driven manner, while the multidimensional model of access, street-level bureaucracy theory, and a social justice perspective informed the analytical interpretation.

**Results:**

The analysis generated five interconnected themes. The findings indicate that formally equal access to the appointment system does not translate into substantively equal opportunities to obtain care. Differences in digital proficiency, social support, transportation access, and temporal flexibility shape patients’ ability to navigate the system, while social connections may provide additional informal access advantages. Staff exercise discretion to assist patients when formal procedures prove insufficient. However, such assistance may make access dependent on staff members’ willingness and capacity to intervene, as well as on organisational approval and available resources. Support for disadvantaged patients is commonly understood not as an institutional right but as an act of conscience and humanity.

**Discussion:**

Overall, the findings reveal a process of conscience-based repair of structurally unequal access: staff attempt to reduce inequities through individual efforts, while the underlying structural conditions remain unchanged. When equitable access is not institutionally guaranteed, responsibility for compensating for these inequalities is partly transferred to frontline staff. Transparent, consistent, and rights-based institutional arrangements are therefore needed to ensure equitable access.

## 1 Introduction

Today, hospital appointment systems are increasingly managed through digital technologies to facilitate the efficient delivery of healthcare services and the allocation of limited consultation slots. These systems are not merely administrative tools that streamline operational processes; they also constitute a critical stage that patients must navigate before accessing healthcare services. However, digitalisation may transform digital skills into an implicit prerequisite for access, thereby creating new barriers for individuals with limited digital literacy or restricted access to technology (1, 2). Accordingly, access to healthcare cannot be explained solely by physical or geographical proximity to healthcare facilities. Penchansky and Thomas (3) conceptualised access as comprising five dimensions: availability, accessibility, accommodation, affordability, and acceptability. Among these, accommodation refers to the extent to which healthcare services are organised to meet patients’ circumstances. Consequently, appointment booking methods, operating hours, and communication channels play an important role in determining access to healthcare.

Türkiye’s Central Physician Appointment System (MHRS), introduced as part of the Health Transformation Program, exemplifies this approach (4). By enabling patients to book appointments via telephone, the internet, and mobile applications, MHRS has contributed to reducing hospital queues as well as the time and costs associated with accessing healthcare services. Previous studies have generally reported high levels of user satisfaction with the system (5–7). Nevertheless, the benefits provided by the system are not distributed equally across all segments of society. In a national study involving 19,389 participants, Demirci and Uğurluoğlu (8) found that age, income, educational attainment, region of residence, and frequency of internet use significantly influenced online appointment-booking behavior. The same study also reported that only approximately one-third of participants used the internet to schedule physician appointments. Other studies have similarly shown that MHRS use increases with higher levels of education and income and is more common among urban residents than among those living in rural areas (6). Furthermore, familiarity with the system decreases with increasing age, while difficulties experienced during the appointment booking process become more frequent (9). Individuals living in rural or geographically remote areas also encounter additional barriers arising from limitations in internet and telephone infrastructure (10).

Türkiye has a predominantly publicly financed healthcare system based on General Health Insurance, and both institutions included in this study were public hospitals. Appointment booking through the Central Physician Appointment System (MHRS) is free of charge. However, healthcare received in public hospitals may involve statutory participation fees or other charges depending on insurance and eligibility status. Foreign-national patients may also be subject to different coverage and payment arrangements depending on their legal and insurance status. Therefore, references to payment in participants’ accounts should be understood as healthcare-related charges encountered during the care process rather than fees for booking an MHRS appointment.

The ease of using a system does not necessarily mean that patients are able to access healthcare services in practice. Difficulties in obtaining appointments, long waiting times in certain outpatient clinics, and insufficient numbers of physicians may limit the effectiveness of the system (11). Longer waiting times reduce patient satisfaction (12), and the performance of the appointment system varies across hospitals and regions (13). These findings suggest that while digital appointment systems facilitate access for some individuals, they may simultaneously perpetuate existing disadvantages for others.

Existing research has primarily focused on patients’ experiences and the overall performance of appointment systems. In contrast, the frontline staff responsible for implementing these systems in everyday practice have received limited scholarly attention. Yet these employees are more than administrative personnel operating a digital platform; they represent the first point of contact between patients and the healthcare system. Studies conducted in Türkiye have shown that the attitudes, knowledge, and helping practices of registration and call-centre staff influence patients’ satisfaction with the appointment process (14). Lipsky’s (15) theory of street-level bureaucracy provides an important framework for understanding the role of these personnel. According to Lipsky, frontline public service workers exercise a degree of discretion when translating general rules into everyday practice. This discretion can influence who receives services, how they receive them, and to what extent they benefit from them. Previous research has shown that such discretion may sometimes operate in favour of disadvantaged individuals, while at other times unintentionally working to their disadvantage (16). Appointment registration staff are therefore important actors who directly observe inequities within the system and attempt to address them under conditions of limited resources.

This study is informed by three theoretical perspectives that operate at complementary levels of analysis rather than as competing alternatives. The multidimensional model of access (3) serves as the descriptive-analytic instrument of the study: it provides the vocabulary for identifying which dimensions of access, namely availability, accommodation, and affordability, are failing according to participants’ accounts (research question 1). Street-level bureaucracy theory (15) constitutes the primary explanatory lens, because the central object of this study is the frontline worker; it directs analysis to how registration staff translate scarce resources, rigid rules, and limited capacity into discretionary practice, and to the conditions under which they intervene (research question 2). The social justice perspective provides the normative–evaluative frame through which these practices are interpreted, distinguishing formal equality from substantive equity and institutional rights from conscience-driven assistance (research question 3) (17, 18). Operationally, the three perspectives were therefore not applied to the data in parallel but sequentially: the access model guided the identification of inequities in the system, street-level bureaucracy guided the analysis of staff responses to those inequities, and the social justice perspective guided the interpretation of the moral and institutional implications of those responses.

Despite this, discussions of social justice in healthcare have largely centred on physicians and policymakers. Similarly, studies of MHRS in Türkiye have focused primarily on patients’ perspectives, quantitative satisfaction surveys, and administrative performance indicators (5, 6, 13, 14). To the best of our knowledge, qualitative research examining how hospital appointment registration staff perceive inequities within the appointment system and how they respond to these inequities remains extremely limited in Türkiye.

The aim of this study was therefore to examine how hospital appointment registration staff perceive inequities in patients’ access to the appointment system, how they respond to these inequities by exercising discretion when formal procedures fail to meet patients’ needs, and how they understand equality, equity, and entitlement. Accordingly, this study addresses the following research questions:

- How do hospital appointment registration staff perceive inequities in patients’ access to the appointment system?
- How do appointment registration staff exercise discretion when formal appointment rules fail to meet patients’ needs?
- How are equality, equity, and entitlement understood in appointment registration staff’s accounts?

## 2 Materials and methods

### 2.1 Study design

A qualitative research design grounded in a constructionist epistemological position was adopted. In this study, participants’ accounts were understood not as direct reflections of an external reality but as meanings constructed through their institutional roles and experiences. This design was considered appropriate because the research questions focus on how appointment registration staff perceive access inequities, exercise discretion, and understand equality, equity, and entitlement. These phenomena were therefore approached as contextually situated meanings rather than as fixed variables that could be examined independently of participants’ institutional experiences. A qualitative approach enabled an in-depth examination of how frontline staff interpret and make sense of these issues. The exploratory nature of the research questions further supported this choice.

Within this qualitative design, the data were analysed using reflexive thematic analysis (19, 20). Reflexive thematic analysis was selected because of its theoretical flexibility, its compatibility with a constructionist epistemological position, and its suitability for identifying patterned meanings across a dataset. It also supports analysis at both semantic and latent levels, which was consistent with the study’s aim of examining participants’ explicit accounts as well as the broader meanings through which institutional responsibility, equality, equity, and entitlement were constructed (19, 20). In line with this approach, themes were understood as interpretive constructions developed through the researchers’ active engagement with the data rather than as entities that simply emerged from the dataset (20).

Coding was conducted predominantly in an inductive and data-driven manner. The multidimensional model of access (3), street-level bureaucracy theory (15), and the social justice perspective were used as sensitising concepts. These perspectives directed analytical attention to issues of access, discretion, and equity without functioning as a predetermined coding framework or constraining the data-driven development of themes (20).

### 2.2 Participants and sampling

The study was conducted in two public hospitals located in a province in the Central Anatolia Region of Türkiye. Participants consisted of appointment registration staff and were recruited using snowball sampling. The first participant was reached through a professional contact of the second researcher, who acted solely as an initial gatekeeper by facilitating access to an eligible appointment registration staff member. This contact did not participate in the study and was not involved in data collection or subsequent recruitment decisions. Participant eligibility was determined solely according to the predefined inclusion criteria. Subsequent participants were recruited through referrals, with particular efforts to reach eligible staff working across different outpatient clinics and registration units. This strategy was intended to reduce dependence on a narrow social network and to capture a wider range of appointment registration experiences within the two institutions. Although overlapping social networks could not be entirely avoided, recruitment sought variation across as many different units as possible. The inclusion criteria were: (a) currently working in a hospital appointment registration unit; (b) having at least 1 year of professional experience; and (c) providing voluntary consent to participate. A total of 20 staff members participated in the study.

The two institutions differed in their organisational characteristics. Hospital A was a city hospital serving both the province and patients from neighbouring provinces and employed approximately 30 appointment registration staff distributed across outpatient clinics and registration units. Hospital B was a university research hospital employing six appointment registration staff. Seventeen staff members from Hospital A and three from Hospital B participated, corresponding to approximately 56.7 and 50% of the appointment registration workforce in each institution, respectively. Recruitment sought variation across outpatient clinics and registration units within these two hospitals rather than statistical representation of hospitals across Türkiye.

Snowball sampling within a limited number of institutions may nevertheless recruit participants from overlapping social networks and may therefore limit the range of perspectives represented in the dataset. Efforts to recruit staff from different units were intended to partially reduce this risk but could not eliminate it entirely. Data collection was concluded not on the basis of data saturation, but because the dataset was considered sufficiently rich and varied to address the research questions within the bounded institutional context and the accessible pool of appointment registration staff.

To preserve anonymity, participant identification codes (K1–K20) were assigned independently of institutional affiliation. The codes do not correspond to institutional order and cannot be linked to a specific hospital in the reporting of findings. Descriptive characteristics are presented as aggregated distributions rather than individual participant profiles (Table 1). No directly identifiable information, such as names or contact details, was collected.

**Table 1:** Distribution of participants’ descriptive characteristics (*N* = 20).

Characteristic	Category	*n*
Age group	20–29 years	7
	30–39 years	12
	≥40 years	1
Gender	Female	18
	Male	2
Professional experience	1–3 years	5
	4–7 years	3
	≥8 years	12
Work unit	General outpatient registration units	11
	Specialised registration units (diagnostic units and specialist outpatient clinics)	6
	Central patient admission/appointment confirmation	3

### 2.3 Data collection

Data were collected between 27 April and 1 June 2026 using a semi-structured interview guide developed by the researchers on the basis of the literature on social justice and access to healthcare. The interview guide consisted of open-ended questions exploring participants’ professional experiences, their observations of patient groups experiencing difficulties in accessing appointments, their perceptions of equity within the appointment system, and their suggestions for improvement.

The interview guide was piloted with the first two participants, who were asked whether the questions were clear and understandable. As no substantial modifications were made following the pilot, these two interviews were retained in the final dataset.

Interviews were conducted face-to-face, with the majority carried out by the second researcher. To ensure confidentiality, interviews took place in a quiet and secluded area of the hospital cafeteria where conversations could not be overheard. No supervisors or colleagues were present nearby. Interviews were conducted during working hours and lasted approximately 20–30 min. Repeat interviews were not undertaken. With participants’ consent, all interviews were audio-recorded and subsequently transcribed verbatim.

Both the interviews and the analysis were conducted in Turkish. Quotations were translated into English by the first researcher and subsequently checked against the original Turkish transcripts by the second researcher, who had conducted the interviews, to ensure semantic and contextual equivalence. Culture-specific expressions were translated functionally while preserving their original meaning.

### 2.4 Data analysis

The analysis was conducted by two researchers with expertise in social work and health management, respectively. This combination shaped the analytical perspective of the study. The social work perspective drew attention to structural disadvantage, discretion, and moral reasoning in frontline practice, whereas the health management perspective supported the interpretation of appointment systems as organisational infrastructures.

The data were analysed using reflexive thematic analysis with the support of MAXQDA qualitative data analysis software (20). The first researcher coded the entire dataset in MAXQDA. The second researcher independently analysed selected information-rich transcripts, coding quotations and candidate themes to generate alternative interpretations. Rather than evaluating coding accuracy or intercoder agreement, these interpretations served to facilitate collaborative reflexive dialogue. Coding proceeded iteratively across the entire dataset. Candidate themes were developed and refined through continual consideration of both the coded extracts and the dataset as a whole.

This process was organised around three analytical meetings. During the first meeting, coding examples and alternative interpretations of the data were discussed. During the second, candidate themes and their central organising concepts were reviewed and refined. During the third, the relationship between the themes and the social justice perspective was examined, leading to the development of the final analytical narrative.

To enhance the transparency and methodological rigour of the analytical process, the full coding structure and its development were documented in the MAXQDA project file. Coded extracts were continually re-examined against the dataset as a whole, and candidate themes were iteratively developed and refined. The reflexive procedures described above were used to explore alternative interpretations and challenge the researchers’ assumptions.

## 3 Results

### 3.1 Participant characteristics and research context

A total of 20 appointment registration staff working in the appointment registration units of two public hospitals participated in the study: 17 from Hospital A and 3 from Hospital B. Regardless of whether patients obtain their appointments in advance through the national Central Physician Appointment System (MHRS) or in person at the hospital, they must interact with appointment registration staff in order to use their appointments. Consequently, irrespective of the appointment booking channel, appointment registration staff constitute the common point of contact through which all patients pass during the appointment process and are therefore in a position to directly observe difficulties in access.

The two hospitals organise this registration function differently. In Hospital B, registration is centralised at a single registration desk located at the hospital entrance, and consequently only a limited number of registration staff are employed. In contrast, Hospital A assigns a separate registration desk and staff member to each outpatient clinic, with patients completing their registration directly at the relevant clinic desk, resulting in the employment of a substantially larger number of appointment registration staff. The asymmetry in participant numbers therefore reflects this structural organisational difference between the two institutions rather than a limitation arising from the sampling strategy.

The study was not designed to compare the two hospitals. Instead, the analysis was conducted using an integrated dataset encompassing both the shared and the divergent experiences of appointment registration staff working across the two institutions. The major themes were represented in accounts from both hospitals; however, because of the non-comparative study design and the unequal sample sizes across institutions, no systematic between-hospital comparison was undertaken.

To preserve participants’ anonymity, descriptive characteristics are presented as aggregated distributions rather than individual participant profiles (Table 1). The majority of participants were women, and more than half had eight or more years of professional experience.

The reflexive thematic analysis generated five interconnected themes. Collectively, these themes suggest that limited appointment capacity interacts with unequal digital, social, geographical, and temporal resources to produce uneven opportunities for access. The resulting gap in access is then addressed through the limited discretion and conscience-driven efforts of frontline staff. The central analytical claims, subthemes, and representative quotations for each theme are presented in Table 2.

**Table 2:** Themes, central analytical claims, and subthemes.

Theme	Central analytical claim	Subthemes	Representative quotation
T1. Falling outside the system’s assumed user profile	The appointment system assumes as its normative user an individual with digital literacy, social support, transportation resources, and temporal flexibility; patients who do not fit this assumption experience greater difficulties in accessing care.	Digital competence becoming a prerequisite for access The accumulation of geographical and economic disadvantage Strategic use of the digital system	*“Older people cannot manage this. Even my own mother and father cannot get an appointment.”* (P12)
T2. Scarce capacity becoming competition among patients	Insufficient physician availability and appointment capacity, together with technical and procedural rules, create a system in which patients compete for scarce appointments, shifting responsibility for limited capacity onto patient behavior.	Rapidly exhausted appointment slots Technical problems and rules governing appointment use Institutional inadequacies turning into conflict between patients and staff	*“If a patient misses an appointment, they cannot book another one for 15 days.”* (P5)
T3. Social Connections as Informal Access Advantages	Although the appointment system is formally open to all users, social connections and perceived management-related channels may create informal advantages in accessing scarce appointments.	Personal and institutional connections The intertwining of formal rules and informal access channels	*“Just because I do not know anyone, does that mean I cannot receive care?”* (P18, recounting a patient’s words)
T4. Discretion humanises the system but cannot repair it	Staff attempt to fill institutional gaps through personal effort and interpersonal relationships, but these efforts are constrained by hierarchy, capacity, and workload.	Helping by relaxing formal rules Dependence on physician and managerial approval Bearing the emotional burden of access problems	*“I can never make the decision on my own; I have to ask them.”* (P18)
T5. Constructing equity as conscience-driven assistance rather than an institutional right	Differential treatment of disadvantaged patients is understood not as an institutional right governed by explicit criteria but as a form of assistance dependent on the conscience of appointment registration staff.	Different meanings attributed to equality Entitlement and concerns about misuse Moving from individual assistance towards institutional arrangements	*“This is not about justice; it is about conscience.”* (P7)

### 3.2 Theme 1: falling outside the system’s assumed user profile

A prominent pattern recurring across the dataset in participants’ accounts was that the appointment system implicitly assumes a particular user profile. This profile can be characterised as a user who has digital literacy, can rely on support from a family member, can easily travel to the hospital, and has temporal flexibility. Participants’ accounts indicated that users with these characteristics were placed in an advantageous position within the system, whereas patients lacking these resources experienced greater difficulties in access. Staff most frequently identified older patients among these groups, describing them as unable to use digital appointment channels independently and as being able to navigate the system only with the assistance of a family member. At times, these observations intersected with participants’ personal experiences. One participant reported that their own parents were also unable to obtain appointments (P12). The perception that digitalisation does not make access easier for everyone, but instead provides selective benefits, further supported this pattern:

*“It has become easier for those who know how to use the system, while the rest continue to experience the same difficulties.”* (P5)

Digital proficiency also influenced patients’ ability to compete for scarce appointment slots. Participants noted that users who enabled notifications, knew when new appointments became available, and understood how to secure cancelled appointments were better positioned to obtain appointments quickly:

*“Patients who enable notifications can immediately book an appointment when a cancelled or unfilled slot becomes available.”* (P15)

This finding suggests that digital proficiency not only reduces barriers for some users but also creates unequal opportunities to act quickly within a capacity-constrained appointment system. Disadvantage was not limited to digital skills but appeared to intersect with geographical and economic factors. In the province where the study was conducted, transportation difficulties between the central district and surrounding districts and villages could make procedures that formally operate in the same way for everyone unequal in practice. One participant illustrated this through the practice of obtaining a queue number:

*“Patients living in the city centre can get a queue number at 5:30 a.m., go home, and come back around 8:00, whereas patients coming from villages cannot go back, so they get their number and have to wait.”* (P20)

Participants also described financial barriers affecting some foreign-national patients. Although MHRS appointment booking itself is free of charge, participants referred to payments reportedly encountered during subsequent stages of healthcare delivery. These payments were perceived as creating an additional financial burden: *“They have to pay for registration, and then they have to make further payments at later stages… because they have no choice, they still have to be examined”* (P19). This quotation reflects the participant’s perception of healthcare-related charges. The specific fee arrangements described were not independently verified through institutional documents and should therefore not be interpreted as a general description of payment rules for all foreign-national patients.

An analytically notable finding was the variation in how staff explained disadvantage. While some accounts attributed the problem to system design and structural conditions, others attributed it to patients’ lack of knowledge: *“Patients are uninformed. Some don’t even know what the appointment line is”* (P7). Framing access problems in terms of patients’ lack of knowledge can be interpreted as individualising institutional responsibility. This variation suggests that the ways in which staff define disadvantaged groups constitute a finding in their own right.

### 3.3 Theme 2: scarce capacity becoming competition among patients

Participants’ accounts suggested that the root of access problems lies not in isolated disruptions but in the transformation of scarce capacity into competition among patients. Insufficient physician availability and limited appointment capacity constitute the basis of this competition:

*“We don’t have enough physicians, and the number of available appointments is very limited. Patients cannot receive the care they want or deserve.”* (P10)

In participants’ accounts, scarcity was most clearly reflected in the rapid exhaustion of appointment slots in high-demand outpatient clinics during the first minutes after they became available: *“Dermatology appointments fill up very quickly; even by the time I arrive, they’re already gone.”* (P19). This scarcity was further compounded by technical problems. One participant reported issues such as duplicate appointments appearing in the system, appointments not appearing at all, and cancellation notifications failing to reach patients (P7).

A particularly significant finding within this theme was that insufficient capacity becomes reframed as an issue of patient behavior through procedural rules. The restriction imposed on patients who miss their appointments provides a clear example:

*“If a patient misses an appointment, they cannot book another one for 15 days.”* (P5)

Such rules are intended to ensure that the limited number of appointment slots is used more efficiently. However, according to participants, these practices also create new problems for some patients. When patients are unable to obtain appointments, their frustration is often directed towards appointment registration staff. Consequently, the effects of insufficient hospital capacity emerge and are negotiated through the everyday interactions between patients and appointment registration staff.

### 3.4 Theme 3: social connections as informal access advantages

Beyond differences in digital proficiency, participants described social connections as another source of unequal access within a formally standardised system. Several participants suggested that knowing someone within the hospital could facilitate appointment access or help patients navigate existing constraints more easily:

*“If you know someone, you don’t experience any problems at all.”* (P3). This situation also creates a moral burden for staff. For example, one participant recalled a question posed by a patient that vividly conveyed this burden:

*“A patient once said to me, ‘Just because I don’t know anyone, does that mean I can’t receive care?’ Honestly, that really affected me.”* (P18)

One participant also suggested that a proportion of appointment slots could be allocated in response to requests associated with hospital management (P19). This claim is based on the account of a single participant and was not verified through institutional records. Nevertheless, it is analytically meaningful because it reflects staff perceptions that formal rules may become intertwined with informal channels of access. Consequently, participants’ accounts suggest that social connections may create informal advantages within an appointment system that is formally open to all users.

### 3.5 Theme 4: discretion humanises the system but cannot repair it

When formal rules failed to meet patients’ needs, staff attempted to facilitate access by drawing on their personal relationships and their limited scope for discretion. One of the most frequently described practices involved requesting an additional appointment slot from a physician whose schedule was already full:

*“We asked the physician, and the physician agreed.”* (P6)

In some situations, staff also provided assistance beyond their formal responsibilities. One participant explained that when the system could not provide a solution, they called the appointment call centre using their own mobile phone (P5). The perceived urgency of a patient’s situation further reinforced such actions. One participant described contacting a physician directly in an urgent case to ensure that the patient was examined (P18). However, this scope for discretion was not autonomous; it remained closely dependent on the approval of physicians and hospital management:

*“I can never make the decision on my own; I have to ask them.”* (P18)

Heavy patient volume, formal rules, and workload further constrained this discretionary space:

*“We can’t call physicians for every patient.”* (P16)

Discretionary efforts also had an emotional dimension. Expressions of gratitude served as a meaningful source of recognition for staff, whereas when assistance could not be provided, appointment registration staff often became the first target of patients’ frustration:

*“Hearing someone say, ‘May God bless you,’ means a lot to us… but when a physician refuses to open an additional appointment slot, patients take it out on us. They get angry, they shout, and we end up feeling bad.”* (P20)

Thus, staff attempted to bridge institutional gaps through personal effort and interpersonal relationships. However, these efforts were insufficient to address the structural problems embedded in the system, and the emotional burden of access problems was borne largely by appointment registration staff.

### 3.6 Theme 5: constructing equity as conscience-driven assistance rather than an institutional right

Participants’ accounts indicated that equality was understood in different ways, ranging from the application of the same rules to everyone to making adjustments according to differing needs. One group of participants regarded applying identical rules to everyone as equality and therefore viewed the current system as already equitable: *“I think everyone is already treated equally.”* (P11). In contrast, another recurring perspective across the dataset was that applying the same rules to patients with different circumstances does not produce equality in practice:

*“I don’t think that’s really possible. People’s circumstances, health conditions, and ages are not the same.”* (P1)

The central finding of this theme was that needs-based differential treatment was understood not as an institutional right or a standardised arrangement, but as a form of assistance left to the individual moral judgement of staff. Even participants who supported giving priority to disadvantaged patients framed their views primarily in terms of conscience, humanity, and helping rather than in the language of rights. This pattern was evident across several participants’ accounts:

*“Helping very old, frail, disabled, or pregnant patients is a humanitarian responsibility. There is nothing open to debate about that, because this is not about justice; it is about conscience.”* (P7)

*“Of course, everyone in a hospital needs help, but… I help every patient I can, as much as I am able.”* (P15)

This discourse shifts equity from the domain of institutional responsibility to the moral discretion of individual staff members. Concerns about the potential misuse of priority arrangements further revealed a lack of trust in such practices: *“This would be open to a great deal of abuse.”* (P3). Likewise, one participant’s objection to the priority reportedly given to hospital employees (P19) highlighted the ambiguity surrounding the criteria by which individuals are regarded as entitled to priority.

Participants’ recommendations clustered around two broad areas. The first focused on supply-side measures, particularly increasing the number of physicians and expanding appointment capacity:

*“Increasing the number of physicians would prevent these difficulties.”* (P4)

The second focused on institutionalising access support through measures such as establishing appointment assistance units within hospitals and strengthening integration with family medicine:

*“There could be an appointment assistance unit within the hospital.”* (P9)

The common thread across these recommendations was the transfer of access support from its current reliance on individual conscience and informal assistance to formal institutional arrangements.

## 4 Discussion

This study examined how hospital appointment registration staff perceive inequities within the appointment system and how they respond to these challenges. The findings indicate that although the system appears to be equally accessible to everyone, patients do not enter it under equal conditions. Digital skills, social support, transportation resources, temporal flexibility, and personal connections are not equally distributed among patients. In addition, appointment capacity is limited. Appointment registration staff attempt to bridge the gap between patients’ needs and the opportunities provided by the system through personal effort and the exercise of discretion. Although these efforts enable some patients to access healthcare services, they also make access dependent on staff members’ willingness and available opportunities, without addressing the underlying inequities embedded within the system. Taken together, these findings can be interpreted through three complementary analytical perspectives. Penchansky and Thomas’s multidimensional model identifies where mismatches between patients and healthcare services occur, street-level bureaucracy helps explain how frontline staff respond to these mismatches in everyday practice, and the social justice perspective provides a normative basis for evaluating whether these arrangements produce equitable access.

Importantly, these dimensions appear to interact rather than operate independently. Limited availability intensifies inequalities in accommodation because when appointment slots are scarce, the ability to monitor the system and respond quickly becomes increasingly consequential. Similarly, geographical accessibility interacts with accommodation because obtaining an appointment digitally does not eliminate the transportation and temporal constraints involved in actually using that appointment. Affordability may likewise intersect with accessibility when travel or healthcare-related costs place additional burdens on patients who already face geographical disadvantage. The findings therefore suggest that technical access to an appointment system should not be equated with effective access to healthcare. A service may be formally available through the same digital interface to all patients while remaining unequally usable because the resources required to convert that opportunity into actual care are unevenly distributed.

These findings are consistent with studies showing that digital health technologies may perpetuate or even exacerbate existing inequities (1, 2). In Türkiye, the use of online appointment systems varies according to age, income, educational attainment, and region of residence (8). Utilisation increases with higher levels of education and income and is more common in urban than in rural areas (6). Familiarity with the system decreases with increasing age, while the difficulties experienced during appointment booking become more pronounced (9). Individuals living in rural areas also face additional barriers related to internet access, telephone infrastructure, and transportation (10).

Evidence from countries with different healthcare financing and organisational arrangements indicates that this tension between the benefits of digitalisation and the risk of widening inequalities is not unique to Türkiye. A recent scoping review found that although digital health technologies can improve access and efficiency, their benefits may be disproportionately concentrated among populations with greater socioeconomic and digital resources, thereby reinforcing existing inequalities (21). In England’s NHS, an analysis covering 6,356 primary care providers found that greater socioeconomic deprivation was significantly associated with lower utilisation of both the NHS App and online primary care services (22). Similarly, Swedish research has identified a pro-rich distribution in digital primary care utilisation, with education and country of birth contributing to socioeconomic inequalities in digital contacts (23). In the United States, patient portal use has been shown to be lower among socioeconomically disadvantaged and uninsured populations and among those living in areas with poorer broadband access (24). Recent Australian evidence likewise indicates that although telehealth policies strongly emphasise improved geographical access, affordability, digital inclusion, and culturally appropriate access remain less consistently addressed (25). Taken together, these studies suggest that information and communication technologies can expand opportunities for healthcare access while simultaneously creating new mechanisms through which pre-existing social, economic, and digital inequalities shape who is able to benefit from those opportunities.

The contribution of this study lies in demonstrating how these inequities are observed and interpreted by appointment registration staff during service delivery. While some participants attributed access problems to the structure of the system, others emphasised patients’ lack of knowledge. Explaining access problems in terms of patients’ lack of knowledge may shift attention away from institutional responsibility. Under such a perspective, patients are expected to adapt to the system rather than the system being adapted to patients. The multidimensional access perspective complicates such an individualised explanation. Difficulty navigating a digital appointment system may appear to be a deficit in patient knowledge, yet the same difficulty can also indicate a mismatch between the way services are organised and the capabilities, resources, and circumstances of the population expected to use them. This distinction is important because locating the problem primarily in the patient directs interventions towards education and adaptation, whereas locating it in the patient-system relationship also requires organisational changes.

The interaction between availability and accommodation is particularly important for interpreting the findings on appointment scarcity. Previous studies have shown that user-friendly appointment systems do not necessarily guarantee actual access when physician availability is insufficient, appointment slots are difficult to obtain, and waiting times are prolonged (11–13). The present findings extend this literature by suggesting that scarcity changes the function of the digital appointment system itself. Under conditions of limited capacity, the system does not simply provide a convenient mechanism for booking care. It also becomes a mechanism through which scarce appointment opportunities are distributed. Consequently, organisational characteristics such as when appointments are released, how notifications operate, and how quickly users can respond become increasingly important. Improvements in digital usability may therefore have limited equity effects if underlying availability remains constrained. From Penchansky and Thomas’s (3) perspective, deficits in availability can amplify mismatches in accommodation rather than functioning as an independent access problem.

The restriction imposed on patients who miss appointments illustrates a similar interaction between service capacity and accommodation. Such rules can be understood as organisational attempts to protect scarce appointment capacity. However, their effects depend on patients’ ability to conform to the assumptions embedded in the rule. Transportation difficulties, caregiving responsibilities, changes in health status, and failures in notification delivery may reduce some patients’ capacity to attend or cancel an appointment within the expected timeframe. A formally identical rule may therefore impose unequal consequences on individuals with different levels of temporal, social, and geographical flexibility. This reinforces a central implication of the multidimensional access model: equal procedures do not necessarily produce equal access when the fit between service organisation and users’ circumstances differs across social groups.

Digital proficiency and social connections represent different mechanisms but may also accumulate within the same patient. Digital proficiency can increase the ability to identify and act on formally available appointment opportunities, whereas social connections may provide access to informal channels of assistance. When these resources coexist with geographical proximity, transportation options, and temporal flexibility, they may generate cumulative advantages in obtaining scarce services. Conversely, patients who lack several of these resources may experience cumulative disadvantage. The relevant equity issue is therefore not that digitally proficient users are improperly privileged, but that formally equal access opportunities may produce unequal outcomes when patients possess unequal resources for navigating them. This distinction is central to differentiating formal equality from substantive equity.

Although personal connections may facilitate patients’ access to appointments, they also create ethical challenges for staff. Appointment registration staff become the visible face of a system they did not design and are confronted with the frustration of patients who are unable to obtain appointments. Consequently, responsibility for the system’s capacity and access problems is displaced onto frontline appointment registration staff during everyday encounters.

Penchansky and Thomas’s framework helps identify where mismatches in access occur, but it does not fully explain how such mismatches are managed in everyday organisational practice. Lipsky’s (15) theory of street-level bureaucracy addresses this second analytical question. Participants’ practices of requesting additional appointment slots from physicians, contacting the appointment call centre on behalf of patients, or relying on personal connections are consistent with Lipsky’s (15) theory of street-level bureaucracy. According to this perspective, frontline workers exercise discretion under conditions of scarce resources and rigid rules. Appointment registration staff therefore do more than simply implement formal rules; they also determine when intervention is warranted.

This discretion has two dimensions. On the one hand, it makes a rigid system more responsive to patients’ needs. On the other hand, it leaves access dependent on staff members’ willingness to help, their workload, and their relationships with physicians. Consequently, patients in similar circumstances may experience different outcomes depending on the staff member they encounter. This finding is consistent with previous studies showing that frontline discretion may operate either in favour of or against disadvantaged individuals (16). In this sense, discretion functions as an informal mechanism for addressing some of the access mismatches identified through Penchansky and Thomas’s framework. It can temporarily compensate for insufficient availability or poorly accommodated procedures, but it does so at the level of individual encounters rather than by changing the organisational conditions that generated the mismatch.

Conscience-based assistance nevertheless carries a risk that merits explicit attention: because intervention depends on individual moral judgement rather than explicit criteria, it is liable to be selective and may generate new imbalances of its own. The street-level bureaucracy literature suggests that when frontline workers must decide whom to help under conditions of scarcity and without clear standards, they fall back on criteria of worthiness (26), concentrating on the character and perceived deservingness of clients rather than on formal rules when allocating resources (27). Client attributes such as perceived motivation and competence influence workers’ willingness to expend extra time and effort on a particular client (28), and workers may favour clients with whom they feel an affinity, a pattern described as emotional creaming (29). Applied to the present findings, this implies that conscience-based assistance may systematically favour patients who present as cooperative, grateful, or socially articulate, whereas patients who are perceived as demanding, unfamiliar, or less able to elicit empathy (despite equal or greater need) may receive less help. Traces of this selection dynamic are visible in the data: staff rationed interventions (“We can’t call physicians for every patient”, P16), and some expressed concern that explicit priority arrangements “would be open to a great deal of abuse” (P3), indicating that judgements about worthiness operate within everyday discretionary practice. The consequence is that conscience-based repair does not simply fail to correct structural inequity; it superimposes a second layer of inequity, making access dependent not only on patients’ digital, economic, and social resources but also on their capacity to attract staff empathy during brief face-to-face encounters. From the social justice perspective adopted in this study, this risk strengthens the case for institutionalising assistance through explicit, transparent, and documentable priority criteria (17, 18).

Staff members’ capacity to intervene is also limited. The opening of additional appointment slots depends on physician and managerial approval. Heavy workloads make it difficult to make separate efforts for every patient. When all appointment slots are completely filled, staff have no remaining means of intervention. Therefore, although staff may alleviate certain problems on a case-by-case basis, they cannot alter the structure that produces those problems. Furthermore, when assistance cannot be provided, appointment registration staff become the first targets of patients’ anger and consequently bear the emotional burden of structural problems within the system. This is also consistent with Coetzer et al.’s (30) observation that responsibility for resolving system-related problems in digital health applications is often placed on patients and healthcare workers. The authors emphasize that this transfer of responsibility may obscure shortcomings at the health-system level and lead structural problems to be compensated for through individual efforts.

The third analytical perspective, social justice, shifts attention from whether everyone is formally subjected to the same appointment rules to whether patients have substantively equitable opportunities to obtain care. One of the important findings of this study is that assistance provided to disadvantaged patients is framed in terms of conscience and humanity rather than as a right. According to Rawls’s theory of justice, applying the same rules to individuals in different circumstances does not, by itself, ensure justice. Instead, institutions should be organised in ways that improve the situation of the least advantaged (18). Some participants recognised that treating patients with different circumstances in the same way does not produce equality; however, they expressed this understanding through the language of assistance rather than that of institutional responsibility. From a social justice perspective, the central issue is therefore not simply whether staff occasionally assist disadvantaged patients. Rather, it is whether support required to achieve equitable access is guaranteed through institutional arrangements or remains contingent on individual moral judgement. When compensatory support is constructed as kindness, conscience, or personal assistance rather than as an institutional entitlement, equity becomes dependent on who is willing and able to intervene.

Concerns about the potential misuse of priority arrangements also point to the absence of explicit criteria. In the absence of transparent and auditable rules, it becomes difficult to distinguish needs-based prioritisation from favouritism. Priority arrangements should therefore not be abolished but instead be governed by explicit and documentable criteria. Institutionalising such criteria would not require abandoning frontline judgement altogether. Rather, it would place discretion within a transparent framework that specifies when differential treatment is justified by differences in need and makes these decisions open to organisational review. This would help distinguish equity-oriented prioritisation from access based on personal connections or arbitrary favour.

Bringing the three analytical perspectives together provides a more integrated interpretation of the findings. Penchansky and Thomas’s framework identifies multidimensional mismatches in access, Lipsky’s theory explains how frontline workers attempt to manage these mismatches under conditions of scarcity and procedural constraint, and the social justice perspective reveals the limitations of leaving such corrective action to individual discretion. The findings therefore suggest a process that can be described as conscience-based repair of structurally unequal access. Limited availability, geographical barriers, organisational arrangements, and unequal patient resources first generate differential opportunities for obtaining care. Frontline staff then use personal effort and discretion to compensate for some of these mismatches on a case-by-case basis. Although these interventions may enable individual patients to obtain services, they do not alter the conditions that produced the disadvantage. Moreover, because the corrective mechanism is grounded largely in individual moral judgement rather than institutional entitlement, it can itself be inconsistently distributed. Conscience-based repair can therefore mitigate individual exclusion while simultaneously leaving the structural production of inequity intact.

This informal repair may also reduce the visibility of problems within the system. Because staff interventions resolve the problems of some patients, structural deficiencies may become less visible from the institutional perspective. Appointment registration staff therefore function as a buffer that both protects patients from system failures and helps sustain the operation of the system. This is also consistent with recent literature emphasising that achieving equity in digital health requires not only user-friendly design but also supportive infrastructures, non-digital alternatives, and human assistance (31). However, the findings of this study show that when such support is not institutionalised, this function is assumed largely and informally by frontline appointment registration staff. This interpretation is also consistent with the broader international literature showing that digital health equity cannot be achieved through technological availability alone and requires attention to socioeconomic resources, infrastructure, digital literacy, affordability, and organisational support (21, 22, 25).

Based on these findings, capacity should first be increased in high-demand outpatient clinics, and the processes governing the release, allocation, and cancellation of appointments should be made more transparent. Problems related to appointment notifications should be minimised, and restrictions imposed on patients who miss appointments should take account of circumstances such as transportation difficulties, caregiving responsibilities, and changes in health status. These measures address both availability and accommodation by combining increased service capacity with organisational arrangements that are more responsive to differences in patients’ circumstances.

Hospitals could establish appointment assistance units or digital guidance desks. Telephone and face-to-face appointment channels should be maintained, and the system should be designed to accommodate the needs of older adults, people with disabilities, migrants, and individuals with limited digital skills. Staff discretion should also be guided by explicit priority criteria, and discretionary decisions should be documented. In addition, appointment registration staff should be provided with institutional support to help them cope with patients’ reactions and the emotional burden associated with their work. More broadly, equity monitoring should extend beyond overall system utilisation and examine whether appointment access differs systematically according to age, socioeconomic position, place of residence, disability, migration status, and digital resources. Such monitoring would shift responsibility for identifying and addressing access inequities from individual frontline workers towards the institutions that design and govern digital appointment systems.

## 5 Conclusion

This study aimed to examine how frontline appointment registration staff perceive and respond to inequities in access to a digital hospital appointment system. The findings provide clear answers to the three research questions. First, staff perceived inequities as arising from the interaction between limited appointment capacity and unequal digital, geographical, temporal, economic, and social resources. Thus, formal access to the same appointment system did not translate into substantively equal opportunities to obtain care. Second, staff used discretion to mitigate some of these barriers, but their capacity to intervene remained constrained by workload, available capacity, and physician or managerial approval. Third, participants frequently constructed needs-based support through the language of conscience and humanity rather than as an institutional entitlement. Taken together, these findings indicate that inequity in digital appointment access is not solely a technological problem but also an organisational and normative one.

The central conclusion is that when mechanisms for equitable access are not institutionally defined, the task of compensating for unequal access is partly transferred to frontline workers. Such discretionary assistance may help individual patients, but it cannot reliably correct structural inequalities and may itself be inconsistently distributed. A more equitable appointment system therefore requires not only adequate capacity and accessible digital interfaces, but also formal mechanisms that recognise differences in patients’ ability to navigate and use the system. Transparent priority criteria, institutionalised access support, and non-digital alternatives are needed so that access does not depend on personal connections, individual staff discretion, or a patient’s ability to elicit assistance.

Future research should examine patients, frontline staff, and hospital management within the same study and compare appointment systems across different institutional and regional contexts. Combining qualitative accounts with administrative appointment data would also help clarify how the access inequalities identified here operate in practice and whether interventions such as institutionalised appointment assistance reduce them.

### 5.1 Limitations

The findings of this study are based exclusively on the accounts of frontline appointment registration staff and do not include the perspectives of patients, physicians, or hospital managers. Accordingly, the findings represent how staff perceived and interpreted appointment-related inequities and institutional practices rather than independently verified accounts of these processes. Some practices may have been understood differently by other stakeholders, and the absence of multiple stakeholder perspectives limits opportunities for comparison and corroboration. Observational data were also not collected, which limits independent assessment of discretionary practices described by participants. Although an official Ministry of Health document (32) was reviewed to distinguish formally regulated procedures from reported informal practices, claims concerning management-related appointment allocations and fees reportedly charged to foreign-national patients could not be independently verified and should therefore be interpreted as participant accounts rather than established institutional facts.

The study was conducted in two public hospitals in a single province of Central Anatolia, with an uneven distribution of participants across the two institutions. Recruitment was initiated through a professional contact of the second researcher, who acted solely as a gatekeeper by facilitating access to the first eligible participant and was not involved in data collection or subsequent recruitment decisions. Although participant eligibility was determined according to predefined inclusion criteria, beginning recruitment through an existing professional network may have influenced the initial recruitment chain and introduced selection bias. Subsequent snowball referrals may also have recruited participants from overlapping social networks and may therefore have limited the diversity of perspectives represented in the dataset. Efforts to recruit staff from different outpatient clinics and registration units were intended to reduce this risk but could not eliminate it entirely. Because chain-referral recruitment is inherently dependent on social networks, the exact participant recruitment pathway may not be reproducible in another setting, although the recruitment procedure, eligibility criteria, and diversification strategy have been described transparently to support methodological assessment and procedural replication. The study was not designed to compare the two institutions, and the findings should not be interpreted as evidence of institutional differences or similarities.

Although the sample included approximately half of the appointment registration workforce in each participating institution, the two hospitals were not selected to represent the full diversity of hospitals using MHRS across Türkiye.

Regarding transferability, consistent with the constructionist epistemological positioning of the study, no claim is made to statistical generalisability. The moral framing of assistance observed in participants’ accounts, particularly through the language of conscience, humanity, and religious gratitude, may partly reflect socio-cultural norms and meanings specific to this setting, including norms of mutual assistance and potentially paternalistic expectations of care. The language through which frontline assistance is understood, and the prominence of conscience-based accounts, may therefore differ across regions and countries. Accordingly, the study’s central analytical proposition, that failures of equity in digital access systems may be informally repaired through frontline discretion and conscience, is offered as a provisional mid-range proposition rather than a universally generalisable claim. Its relevance and limits in settings with different institutional and socio-cultural characteristics should be examined through comparative research.

## Data Availability

The qualitative interview data are not publicly available because they contain potentially identifiable information and are subject to confidentiality and ethical restrictions. De-identified excerpts or data may be made available by the corresponding author upon reasonable request, where permitted by the relevant ethical and data protection requirements.

## References

[1] Veinot TC Mitchell H Ancker JS. Good intentions are not enough: how informatics interventions can worsen inequality. J Am Med Inform Assoc. (2018) 25:1080–8. doi: 10.1093/jamia/ocy052, 29788380 PMC7646885

[2] Yao R Zhang W Evans R Cao G Rui T Shen L. Inequities in health care services caused by the adoption of digital health technologies: scoping review. J Med Internet Res. (2022) 24:e34144. doi: 10.2196/34144, 35311682 PMC8981004

[3] Penchansky R Thomas JW. The concept of access: definition and relationship to consumer satisfaction. Med Care. (1981) 19:127–40. doi: 10.1097/00005650-198102000-00001, 7206846

[4] Evirgen H Yorulmaz M. Merkezi Hekim Randevu Sistemi (MHRS): bir kamu hastanesi örneği. Avrasya Uluslar Araşt Derg. (2019) 7:432–43. doi: 10.33692/avrasyad.543825

[5] Kıraç R. Hastane randevu sistemlerinin hastalar açısından değerlendirilmesi. Sağlık Akademisyenleri Derg. (2019) 6:189–95.

[6] Pekgör S Eryılmaz MA Solak İ Pekgör A Yaka H Kaya İFK . Evaluation of factors affecting the use of the central physician appointment system. South Clin Istanb Eurasia. (2017) 28:204–11. doi: 10.14744/scie.2017.36855

[7] Yıldızbaşı E Öztaş D Sanisoğlu Y Fırat H Dağ Şeker E Doğusan A . Bir eğitim ve araştırma hastanesinde Merkezi Hekim Randevu Sistemini kullanan hastaların memnuniyet düzeylerinin ölçülmesi. Ankara Med J. (2016) 16:293–302. doi: 10.17098/amj.63878

[8] Demirci Ş Uğurluoğlu Ö. İnternet üzerinden hekim randevusu alma davranışının bireysel özellikler açısından değerlendirilmesi. Hacettepe Sağlık İdaresi Derg. (2022) 25:643–54.

[9] Kördeve MK Uzun B Ünal E. Merkezi hastane randevu sisteminin işleyişi üzerine bir alan araştırması. Uluslararası Sağlık Yönetimi ve Stratejileri Araşt Derg. (2017) 3:52–61.

[10] Kurşun A Kaygısız EG. Merkezi Hekim Randevu Sistemi (MHRS) uygulamalarına yönelik memnuniyet ve erişilebilirlik düzeyinin belirlenmesi. Acıbadem Üniv Sağlık Bilim Derg. (2018) 9:401–9.

[11] Topçuoğlu E Kavak O Kaygın E. Sağlıkta yönetim bilişim sistemi olarak MHRS'nin teknoloji kabul modeli ile analizi. İşletme Bilimi ve Uygul Derg. (2022) 2:1–16.

[12] Kan M Alp S Balçık PY. Bekleme süreleri ve hasta memnuniyeti arasındaki ilişki: şehir hastanesi örneği. Çankırı Karatekin Üniv Sos Bilim Enst Derg. (2025) 16:366–77. doi: 10.54558/jiss.1692053

[13] Akın Özdemir N. E-sağlık uygulaması olan Merkezi Hekim Randevu Sisteminin eğitim ve araştırma hastanelerindeki performansının ölçülmesi: Türkiye Düzey-1 örneği. Yonetim Bilim Derg. (2025) 23:280–309. doi: 10.35408/comuybd.1516468

[14] Polat E Özer K. Merkezi Hekim Randevu Sisteminde hasta memnuniyeti araştırması: Güneydoğu Anadolu'da bir kamu hastanesi. Sci Acad Res. (2024) 3:199–212.

[15] Lipsky M. Street-Level Bureaucracy: Dilemmas of the Individual in Public Services. New York: Russell Sage Foundation (1980).

[16] Brodkin EZ. Policy work: street-level organizations under new managerialism. J Public Adm Res Theory. (2011) 21:i253–77. doi: 10.1093/jopart/muq093

[17] Henry TL Bird AN Candler SG. "Healthcare advocacy and social justice". In: Lu LB Fortuna RJ Noronha CF Sobel HG Tobin DG, editors. Leading an Academic Medical Practice. Cham: Springer (2023). p. 387–400.

[18] Rawls J. A Theory of Justice. Cambridge: Harvard University Press (1971).

[19] Braun V Clarke V. Using thematic analysis in psychology. Qual Res Psychol. (2006) 3:77–101. doi: 10.1191/1478088706qp063oa

[20] Braun V Clarke V. Reflecting on reflexive thematic analysis. Qual Res Sport Exerc Health. (2019) 11:589–97. doi: 10.1080/2159676X.2019.1628806

[21] Badr J Motulsky A Denis JL. Digital health technologies and inequalities: a scoping review of potential impacts and policy recommendations. Health Policy. (2024) 146:105122. doi: 10.1016/j.healthpol.2024.105122, 38986333

[22] Zhang J Gallifant J Pierce RL Fordham A Teo J Celi L . Quantifying digital health inequality across a national healthcare system. BMJ Health Care Inform. (2023) 30:e100809. doi: 10.1136/bmjhci-2023-100809, 38007224 PMC10680008

[23] Wilkens J Thulesius H Ekman B. From office to digital primary care services: analysing income-related inequalities in utilization. Int J Equity Health. (2024) 23:86. doi: 10.1186/s12939-024-02184-6, 38689241 PMC11061960

[24] Perzynski AT Roach MJ Shick S Callahan B Gunzler D Cebul R . Patient portals and broadband internet inequality. J Am Med Inform Assoc. (2017) 24:927–32. doi: 10.1093/jamia/ocx020, 28371853 PMC6259664

[25] Wang S Killedar A Norris S Wilson A. Evaluating health equity in Australian telehealth policies: a policy review. NPJ Digit Public Health. (2026) 1:4. doi: 10.1038/s44482-025-00008-0

[26] Döring M Jilke S. Cream-skimming at the frontline: the role of administrative literacy. Public Adm. (2023) 101:1569–86. doi: 10.1111/padm.12900

[27] Tummers LG Bekkers VJJM Vink E Musheno M. Coping during public service delivery: a conceptualization and systematic review of the literature. J Public Adm Res Theory. (2015) 25:1099–126. doi: 10.1093/jopart/muu056

[28] Guul TS Pedersen MJ Petersen NBG. Creaming among caseworkers: effects of client competence and client motivation on caseworkers’ willingness to help. Public Adm Rev. (2021) 81:12–22. doi: 10.1111/puar.13297

[29] Volckmar-Eeg MG Vassenden A. Emotional creaming: street-level bureaucrats’ prioritisation of migrant clients ‘likely to succeed’ in labour market integration. Int J Soc Welfare. (2022) 31:165–75. doi: 10.1111/ijsw.12510

[30] Coetzer JA Loukili I Goedhart NS Ket JCF Schuitmaker-Warnaar TJ Zuiderent-Jerak T . The potential and paradoxes of eHealth research for digitally marginalised groups: a qualitative meta-review. Soc Sci Med. (2024) 350:116895. doi: 10.1016/j.socscimed.2024.116895, 38710135

[31] Wilson S Tolley C McArdle R Lawson L Beswick E Hassan N . Recommendations to advance digital health equity: a systematic review of qualitative studies. NPJ Digit Med. (2024) 7:173. doi: 10.1038/s41746-024-01177-7, 38951666 PMC11217442

[32] Republic of Türkiye Ministry of Health, General Directorate of Health Information Systems. Merkezi Hekim Randevu Sistemi Çalışma Usul ve Esasları Hakkında Yönerge. [Directive on the Working Procedures and Principles of the Central Physician Appointment System]. Ankara: Ministry of Health (2019).

